# Identification of a heparin-binding protein encoded by *Mbov_0510* gene in *Mycoplasma bovis*

**DOI:** 10.1128/jb.00160-25

**Published:** 2025-08-11

**Authors:** Zhixin Ma, Qi Wu, Hui Yu, Qiao Pan, Tong Liu, Jiuqing Xin, Qingyuan Xu

**Affiliations:** 1 State Key Laboratory for Animal Disease Control and Prevention, Harbin Veterinary Research Institute, Chinese Academy of Agricultural Sciences 111613 , Harbin, China; 2 Institute of Western Agriculture, Chinese Academy of Agricultural Sciences 12661, Xinjiang, China; University of Notre Dame, Notre Dame, Indiana, USA

**Keywords:** *M. bovis*, adhesion, heparin, immunogenicity

## Abstract

*Mycoplasma bovis* is a highly infectious pathogenic microorganism that causes various clinical signs in cattle, including pneumonia, arthritis, and mastitis, often resulting in significant economic losses. The adhesion of *M. bovis* to host cells is a pivotal step in the infection process, which is a complex process involving multiple pathogenic and host proteins. Molecules involved in *M. bovis* adhesion and colonization are widely recognized as important virulence factors and often implicated in the infection and pathogenesis. In this study, the *Mbov_0510* gene of *M. bovis* was cloned, and the protein encoded by this gene was expressed and purified. The rabbit polyclonal antibody against this protein was also produced. This protein was shown to be a surface protein and to react with the *M. bovis*-positive serum. The ability of this protein to adhere to host cells was verified using embryonic bovine lung (EBL) and Madin-Darby bovine kidney (MDBK) cells. Furthermore, the adhesion function of this protein was found to be achieved through interaction with heparin on the host cell surface, and the key region of the protein involved in heparin binding was identified. The conservation of the protein encoded by the *Mbov_0510* gene was also analyzed. The results of this study suggest that the protein encoded by the *Mbov_0510* gene is a heparin-binding surface membrane protein of *M. bovis* associated with infection.

**IMPORTANCE:**

Mycoplasmas lack a cell wall, and the membrane proteins interacting with host cells play essential roles in their infection and proliferation processes. In this study, we identified a membrane protein encoded by *Mycoplasma bovis* that interacts with heparin on the surface of host cells. Heparin is widely distributed in various cells and tissues of the host and serves as a receptor for the infection and invasion of many pathogenic microorganisms. The ability of *M. bovis* to invade multiple tissues may be related to its heparin-binding capacity. The heparin-binding protein identified in this study is valuable for further research on the infection and invasion mechanisms of *M. bovis*.

## INTRODUCTION

*Mycoplasma bovis* (*M. bovis*) is a significant cattle pathogen responsible for respiratory disease, mastitis, arthritis, and various other conditions, leading to considerable global losses in the livestock sector (1, 2). It frequently co-infects with other pathogens, including *Pasteurella multocida*, *Histophilus somni*, *Histophilus somni*, bovine herpesvirus 1, bovine respiratory syncytial virus, bovine viral diarrhea virus, and parainfluenza virus type 3 (3–7). The combination of pathogens under discussion has been demonstrated to worsen disease severity. Furthermore, *M. bovis* has been identified as one of the pathogens that contribute to the bovine respiratory disease complex (BRDC) (8, 9). As a cell-wall-less mycoplasma, *M. bovis* relies on surface membrane proteins to adhere to host cells, colonize tissues, invade cells, establish persistent infections, and evade the immune response.

Pathogen colonization of the host is a complex process that may involve multiple adhesins or attachment mechanisms. The binding targets of bacterial adhesins are typically the components on the host cell surface, including collagen, elastin, fibronectin, plasminogen, laminin, heparin, and platelet-derived growth factor, among others (10, 11). To date, several *M. bovis* adhesins have been identified, including NOX (12), MbfN (13), FBA (14), TrmFO (15), α-enolase (16), MilA (17), P27 (18), LppA (19), LppB (20), VpmaX (21), P26 (22), VSP (23, 24), the *Mbov-0503* encoded protein (25), and the 24 kDa protein (26). These *M. bovis* proteins play a role in colonization and infection by interacting with various cell surface components, including plasminogen, fibronectin, amyloid precursor-like protein 2, heparin, annexin A2, collagen IV, vitronectin, and laminin (19, 27). Among these binding targets of *M. bovis* adhesins, heparin is of particular interest. The spatiotemporal regulation of the chemical structure of the heparin chains modulates their ability to engage a broad spectrum of heparin-binding proteins (28). A variety of pathogens, including hepatitis C virus (10), dengue virus (11), *Mycoplasma hyopneumoniae* (29), *Candida albicans* (30), and SARS-CoV-2 (31), infect cells by interacting with heparin. It has been hypothesized that the affinity of viral surface molecules for heparin may be an important determinant of tissue tropism and pathogenicity, and heparin with specific sulfation patterns may also act as a cell surface receptor mediating viral entry (32). As a widely distributed sulfated glycosaminoglycan, heparin is present in almost all types of tissues and cells at both the extracellular and cellular levels. The association of heparin with *M. bovis* infection of multiple tissues and cells may be implicated.

In 1989, Cardin and Weintraub identified two significant motifs of heparin-binding proteins: [-X-B-B-X B-X-] and [-X-B-B-B-X-X-B-X-], where B denotes a basic amino acid, and X is a hydrophobic amino acid residue. The identification of these motifs has provided a crucial theoretical foundation for subsequent studies on heparin-binding proteins. MbfN and MilA of *M. bovis*, which bind to heparin, are adhesins that possess these two motifs (13, 17). However, not all heparin-binding proteins contain the classic heparin-binding motif. Although the presence of these motifs suggests that a protein might bind to heparan sulfate, at least half of the known heparin sulfate-binding proteins do not contain Cardin-Weintraub sequences (28). Considering the potential role of heparin in the multi-tissue cell invasiveness of *M. bovis*, it can be inferred that there may be multiple heparin-binding proteins with activity. It is important to note that, in addition to proteins with the classic heparin-binding motif, there are likely to be heparin-binding proteins *of M. bovis* that lack this sequence feature.

To identify *M. bovis* adhesins, we previously attempted immunoprecipitation using whole-cell lysates or surface membrane proteins of *M. bovis* with embryonic bovine lung (EBL) and Madin-Darby bovine kidney (MDBK) cells, followed by mass spectrometry analysis to screen for proteins that can interact with host cells. In the present study, a heparin-binding surface protein of *M. bovis* was identified using various biological and immunological methods. The protein encoded by the *Mbov_0510* gene has been observed to bind to EBL or MDBK cells via heparin, with a 63-amino acid region playing a crucial role. Conservation analysis revealed that this protein is conserved in *M. bovis* and has homologs in *Mycoplasma agalactiae*. Notably, this protein lacks the conventional heparin-binding motif.

## RESULTS

### Expression, immunogenicity, and subcellular localization identification of the *Mbov_0510*-encoded protein

**Fig 1:**
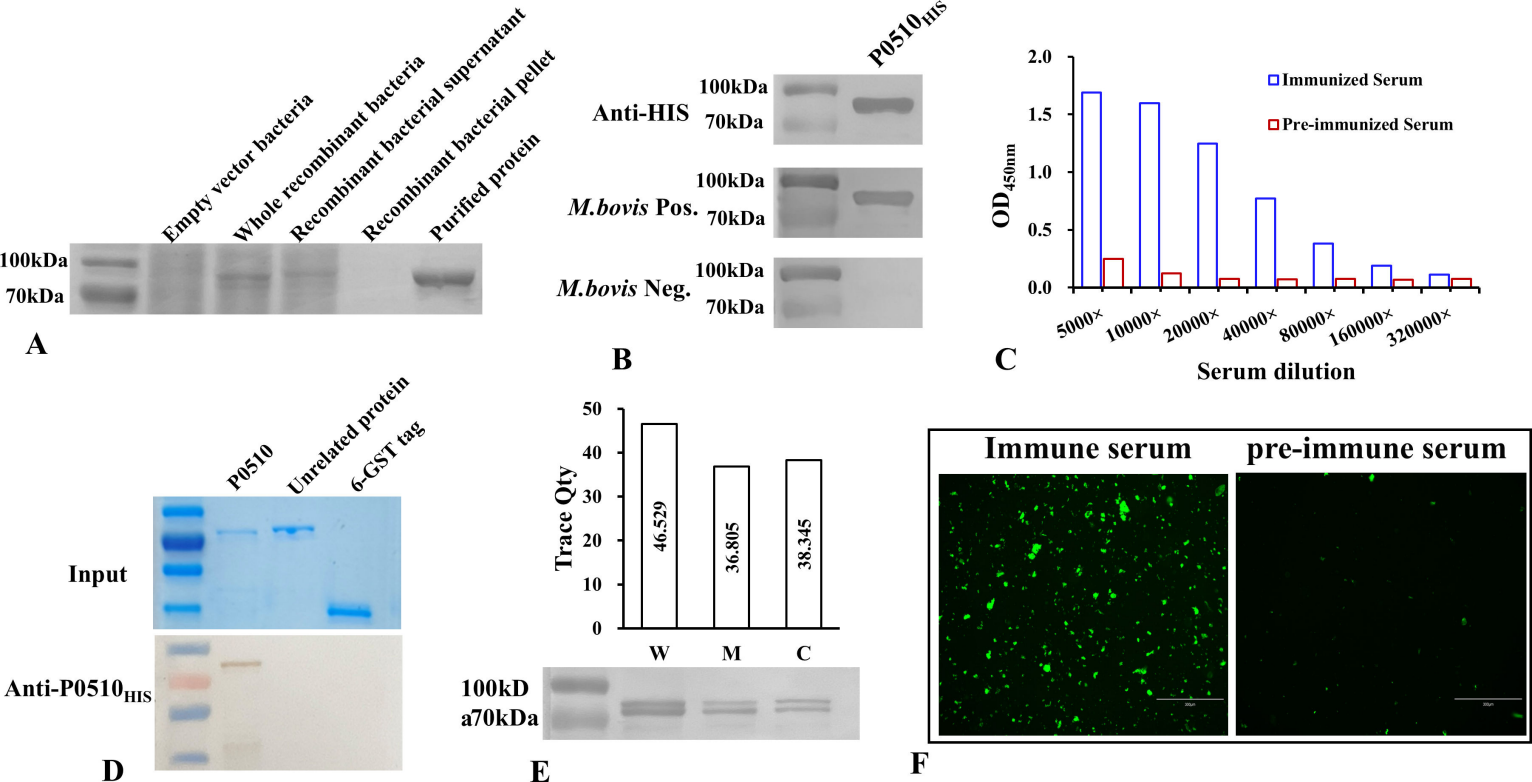
(**A**) SDS-PAGE analysis of induced expression proteins shows that compared with the pET-28a(+) empty vector (lane 1), the *Mbov_0510* recombinant strain exhibits an expected-sized band at the position of 70–100 kDa after induction (lane 2). The expressed protein mainly exists in a soluble form (lane 3), and the target protein is not detected in the cell lysate precipitate (lane 4). The expressed protein can be effectively purified (lane 5). (**B**) The purified protein was transferred to a nitrocellulose (NC) membrane and detected with an anti-HIS tag antibody, revealing a clear band between 70 and 100 kDa. The expressed P0510_HIS_ protein can react with *M. bovis* positive serum but is not recognized by *M. bovis-*negative serum. (**C**) The titer of the rabbit immune serum prepared with the P0510_HIS_ protein was 1:160,000. (**D**) The prepared rabbit anti-P0150_HIS_ serum can specifically recognize the P0510 protein and does not react with unrelated protein or 6-GST tagged proteins. (**E**) Western blot detection results show that the protein encoded by *Mbov_0510* was detected in the whole cell protein, cell membrane protein, and cytoplasmic protein of *M. bovis*. The grayscale analysis results indicate that the expression levels in the cell membrane and cytoplasm are similar. (**F**) Rabbit anti-P0510_HIS_ antibody used for IFA detection of *M. bovis* shows distinct green fluorescence, while the negative serum group exhibits no specific fluorescence.

Using the genome of *M. bovis* strain TJ as a template, the open reading frame of the *Mbov_0510* gene was amplified with specific primers and cloned into the pET-28a(+) vector. The tryptophan codons at positions 299, 516, 566, 628, and 637 in the open reading frame, which were encoded by "TGA" in the mycoplasma, were mutated to "TGG" to construct the prokaryotic expression plasmid. After correct sequencing of the prokaryotic expression plasmid, it was transformed into BL21 (DE3)-competent cells for induced expression, yielding the recombinant protein P0510_HIS_. SDS-PAGE was used to identify the expressed protein, and the results showed that the protein was successfully expressed. The expressed protein was mainly in a soluble form and could be effectively purified (Fig. 1A). The purified protein was identified using a monoclonal antibody against the HIS tag, and a distinct band at 85 kDa was observed (Fig. 1B). Western blot was used to assess the immunogenicity of P0510_HIS_, and it was found that *M. bovis*-positive serum could react with P0510_HIS_, whereas negative serum could not recognize this protein (Fig. 1B). These results indicate that cattle infected with *M. bovis* can produce antibodies against P0510, suggesting that this protein is an immunogenic component of *M. bovis*.

Purified P0510_HIS_ protein was used to immunize Japanese white rabbits to obtain hyperimmune serum against P0510_HIS_. The titer of the serum was determined to be 1:160,000 by enzyme-linked immunosorbent assay (ELISA) (Fig. 1C). The specificity of the rabbit anti-P0510_HIS_ serum was further confirmed by western blot. The results indicated that the serum specifically recognized the P0510 protein and did not react with an unrelated protein and the 6-GST tag (Fig. 1D).

To determine the subcellular localization of the protein encoded by *Mbov_*0510 in M*. bovis*, total-cell proteins, membrane proteins, and cytoplasmic proteins of *M. bovis* were analyzed using rabbit anti-P0510_HIS_ serum. Western blot analysis revealed consistent bands in all three samples, indicating that the protein encoded by the *Mbov_0510* gene is present in the whole cell, cell membrane, and cytoplasm of *M. bovis* (Fig. 1E). Since western blot cannot completely exclude the possibility of cytoplasmic protein contamination in the membrane protein fraction, we further confirmed the surface localization of the *Mbov_0510*-encoded protein in *M. bovis* using indirect immunofluorescence (IFA). After reaction with rabbit anti-P0510_HIS_ serum, *M. bovis* exhibited clear green fluorescence signals under a fluorescence microscope (Fig. 1F), whereas no specific fluorescence signal was observed with pre-immune rabbit serum (Fig. 1G). Taken together, these experiments collectively demonstrate that the protein encoded by *Mbov_0510* is located on the surface of *M. bovis*.

### *Mbov_0510*-encoded protein adheres to EBL and MDBK cells

**Fig 2:**
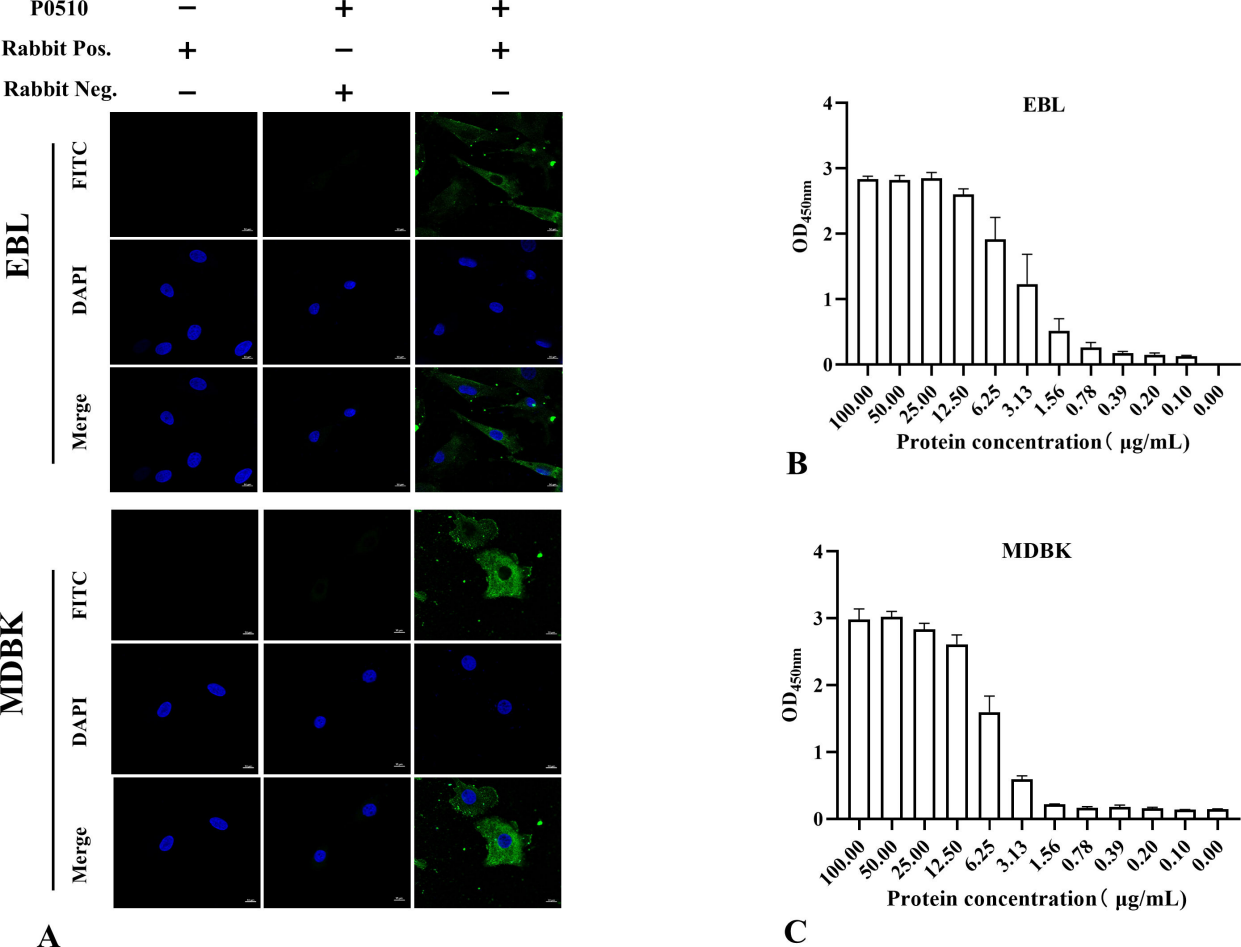
(**A**) Confocal laser scanning microscopy analysis of the adhesion of *Mbov_0510*-encoded protein to host cells. EBL and MDBK cells were incubated with the P0510 protein. Detection with rabbit anti-P0510_HIS_ antibody revealed distinct green fluorescence. No specific green fluorescence was observed in the PBS control group or the negative serum group. (**B**) ELISA confirmed that P0510_HIS_ has an adhesive effect on EBL cell membrane components, with a dose-dependent response reaching saturation at a concentration of 25 µg/mL. (**C**) The interaction of P0510_HIS_ with MDBK cell membrane components was also dose-dependent, with saturation observed at a concentration of 50 µg/mL.

Adhesion of P0510 to EBL and MDBK cells was observed by confocal laser scanning microscopy. After co-incubation of P0510 with EBL and MDBK cells, distinct green fluorescence was detected in both cell types using rabbit anti-P0510_HIS_ serum (Fig. 2A). However, no specific fluorescence was observed in the pre-immune serum group or in the non-recombinant protein group. These results indicate that the protein encoded by the *Mbov_0510* gene can adhere to both EBL and MDBK cells.

To further validate the binding of *Mbov_0510*-encoded protein to EBL and MDBK cells, the ability of the *Mbov_0510*-encoded protein to adhere to the membrane components of EBL and MDBK cells was analyzed using an ELISA method. For the membrane components of EBL cells, saturation of P0510_HIS_ binding was observed at a concentration of 25 µg/mL, and the optical density (OD) value approached background levels after 0.78 µg/mL (Fig. 2B). For the membrane components of MDBK cells, saturation of binding was observed at a concentration of 50 µg/mL, and the OD value approached background levels after 1.568 µg/mL (Fig. 2C). Moreover, the interaction of P0510_HIS_ with the membrane components of both EBL and MDBK cells showed a dose-dependent characteristic.

### *Mbov_0510*-encoded protein interacts with heparin

**Fig 3:**
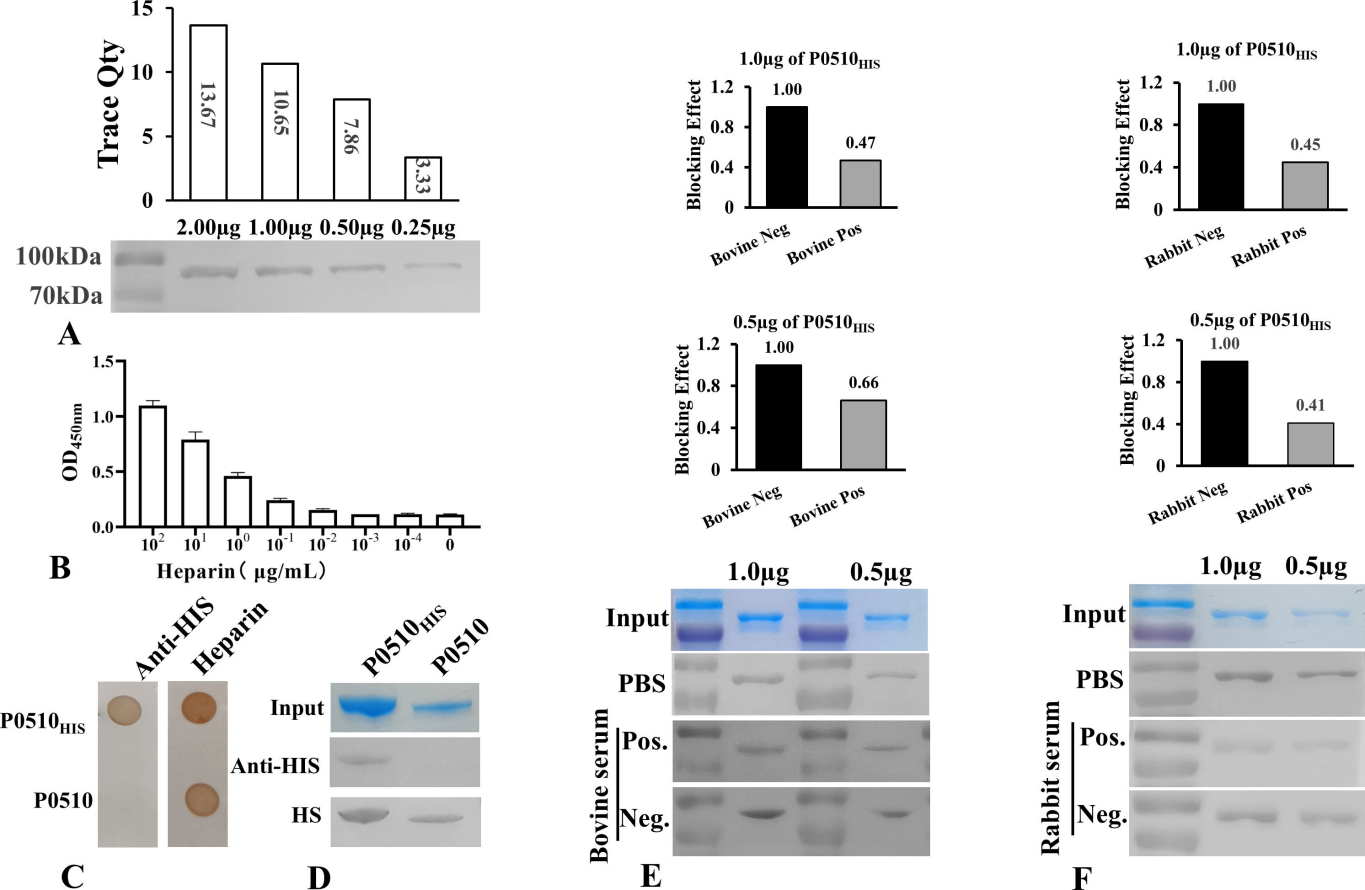
(**A**) Western blot experiments confirmed that P0510_HIS_ at different concentrations can directly interact with heparin. The results of the densitometric analysis showed that the reaction intensity was positively correlated with the concentration of the P0510_HIS_ protein. (**B**) ELISA analysis confirmed that P0510_HIS_ can interact with heparin, and the reaction intensity was positively correlated with the amount of heparin used. (**C**) Dot blot analysis verified that the HIS tag was successfully removed, and the tag had no effect on the binding of the *Mbov_0510*-encoded protein to heparin. (**D**) Western blot further confirmed that the HIS tag was successfully removed, and the fusion of the HIS tag did not affect the interaction between the *Mbov_0510*-encoded protein and heparin. (**E**) Western blot confirmed that *M. bovis* positive serum from bovine could partially block the adhesion of the *Mbov_0510*-encoded protein to heparin. (**F**) Western blot results showed that rabbit anti-P0510_HIS_ serum had a blocking effect on the adhesion of the *Mbov_051*0-encoded protein to heparin.

Heparin is a widely distributed sulfated glycosaminoglycan that is present at both the extracellular and intracellular levels. Many pathogens can interact with heparin on the surface of host cells. To determine whether the protein encoded by *Mbov_0510* could bind to heparin, the interaction between P0510_HIS_ and heparin was analyzed using western blot. The results showed that heparin can bind to P0510_HIS_ protein immobilized on the nitrocellulose (NC) membrane. After staining with DAB substrate, a distinct band appeared at the expected position, and the intensity of the band was found to be proportional to the amount of P0510_HIS_ protein (Fig. 3A). This result indicates that the protein encoded by *Mbov_0510* can bind to heparin on the cell surface.

To further verify the interaction between the protein encoded by *Mbov_0510* and heparin, the P0510_HIS_ protein was coated on an ELISA plate and reacted with biotinylated heparin at different concentrations. The ELISA experiment also confirmed that the P0510_HIS_ protein can bind to heparin in a dose-dependent manner. As the concentration of heparin decreased, the OD values in the corresponding wells of the ELISA plate decreased correspondingly (Fig. 3B).

 Heparin is a negatively charged molecule that typically interacts with positively charged amino acids in heparin-binding proteins. To avoid interference from the HIS tag in determining the binding of the *Mbov_0510*-encoded protein to heparin, the LEVLFQGP sequence was introduced into the fusion protein, and the HVR-3C enzyme was used to remove the HIS tag. The binding of the tag-free protein to heparin was verified by dot blot and western blot analyses. The results indicated that the HIS tag does not affect the interaction between the *Mbov_0510*-encoded protein and heparin. The protein encoded by the *Mbov_0510* gene can bind to heparin, regardless of the presence of the HIS tag (Fig. 3C and D).

### Adhesion inhibition assay for P0510_HIS_ and heparin

To analyze the blocking effect of *M. bovis* positive serum on the adhesion of the *Mbov_0510*-encoded protein, positive serum from cattle naturally infected with the *M. bovis* TJ strain was used to block the interaction between P0510_HIS_ and heparin. P0510_HIS_ protein was transferred onto nitrocellulose (NC) membranes at concentrations of 1.0 and 0.5 µg/mL. Prior to the reaction with heparin, positive serum from cattle infected with *M. bovis* was incubated with P0510_HIS_ protein, with negative serum used as a control. The results showed that serum from naturally infected cattle significantly blocked the binding of P0510_HIS_ to heparin (Fig. 3E). Grayscale analysis of the western blot showed that the binding capacity of P0510_HIS_ at 1.0 and 0.5 µg/mL was reduced by 53 and 34%, respectively, compared to negative serum after blocking with serum from cattle naturally infected with *M. bovis* (Fig. 3E).

The effect of rabbit anti-P0510_HIS_ antibodies on the binding of P0510_HIS_ protein to heparin was analyzed using the same method as that used for *M. bovis*-positive serum. The results showed that anti-P0510_HIS_ antibodies could also inhibit the binding of the protein to heparin (Fig. 3F). The adhesion ability of P0510_HIS_ protein at concentrations of 1.0 and 0.5 µg/mL was reduced by 55 and 59%, respectively, when treated with rabbit anti-P0510_HIS_ antibodies (Fig. 3F).

### Identification of binding regions to heparin for the *Mbov_0510-*encoded protein

**Fig 4:**
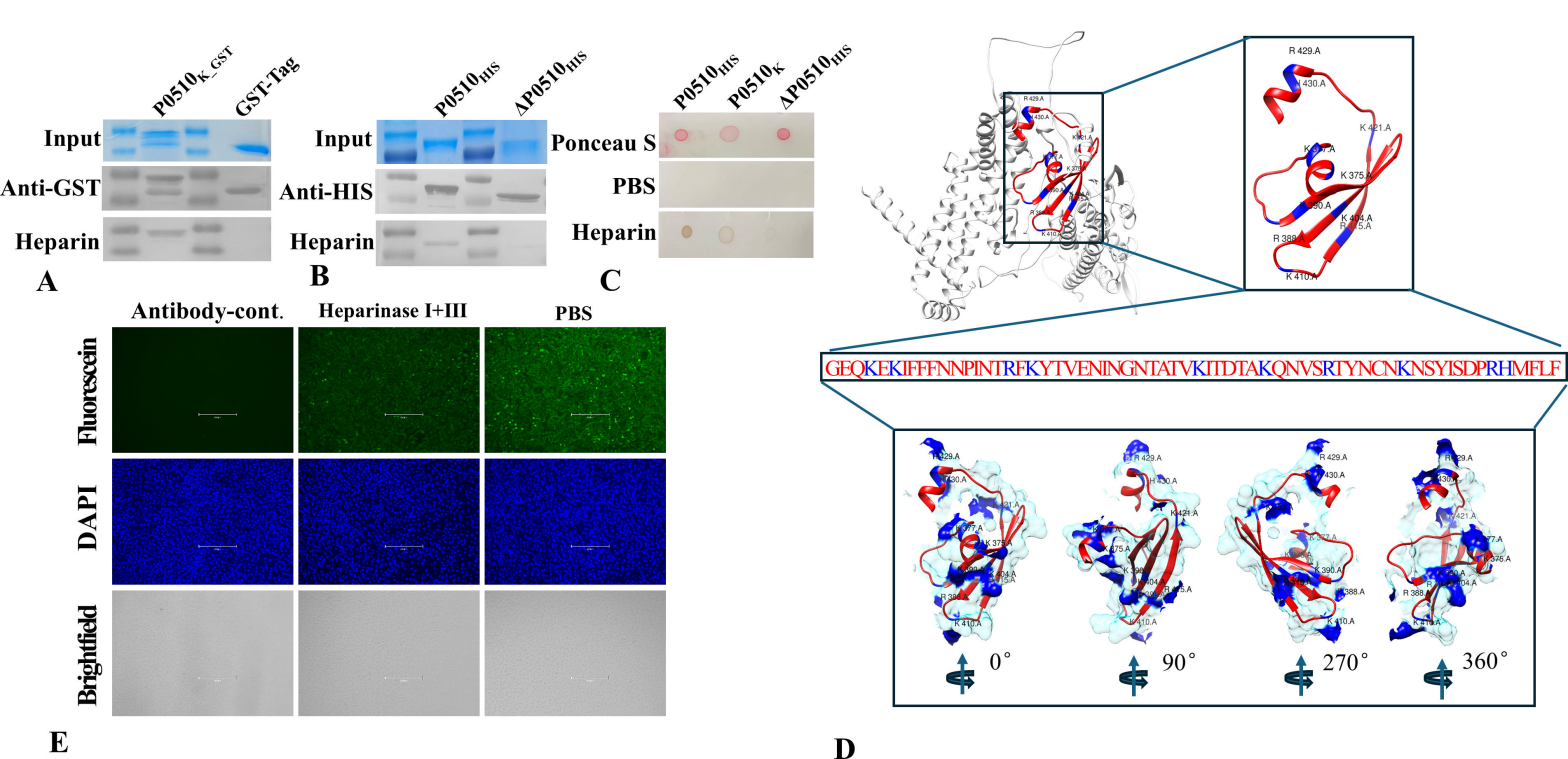
(**A**) The amino acid region 396–458 of the protein encoded by the *Mbov_0510* gene is the minimal region required for heparin binding. (**B**) After deletion of the amino acid region 396–458, the protein encoded by the *Mbov_0510* gene loses its ability to bind to heparin. (**C**) Dot blot results show that both the full-length protein encoded by the *Mbov_0510* gene and its amino acid region 396–458 can bind to heparin, while the mutant of the *Mbov_0510*-encoded protein lacking the amino acid region 396–458 loses the ability to bind to heparin. (**D**) From the tertiary structure perspective, the positively charged amino acids in the amino acid region 396–458 of the *Mbov_0510*-encoded protein are mainly located in alpha helices and beta-sheet structures, and these amino acids are all on the protein surface (blue: positively charged amino acids and their associated surface structures). (**E**) Treatment of MDBK cells with both heparinases I and II significantly reduces the adhesion of P0510_K_ to the cells.

To further analyze the heparin-binding properties of the protein encoded by *Mbov_0510*, a series of truncation expressions or fragment deletions were performed to identify the key region of the protein that reacts with heparin. Western blot analysis revealed that the amino acid region 396–458 of the *Mbov_0510*-encoded protein is the minimal fragment that reacts with heparin, and this region labeled with GST was named P0510_K_GST_ (Fig. 4A). The ΔP0510_HIS_ protein obtained by deleting this region lost the ability to bind heparin (Fig. 4B). To avoid the effect of the structural loss on the western blot results, a dot blot was used to verify the role of the amino acid region 396–458 of the *Mbov_0510*-encoded protein in the interaction with heparin. Under non-denaturing conditions, the intact P0510_HIS_ and P0510_K_ can react with heparin, whereas the ΔP0510_HIS_ protein also lost the ability to react with heparin. This result further confirms that the amino acid region 396–458 of the *Mbov_0510*-encoded protein is the key region for interaction with heparin (Fig. 4C).

Heparin is a large molecule with a significant amount of negative charge. The positively charged amino acids histidine (H), lysine (K), and arginine (R) play an important role in the interaction between proteins and heparin. To analyze the amino acids that may be involved in the binding of the protein encoded by *Mbov_0510* to heparin, this protein was submitted to the SWISS-MODEL online server for structural prediction and analyzed the distribution of positively charged amino acids (basic amino acids) within the 396–458 region of the protein in its tertiary structure. The results showed that there are 10 positively charged amino acids in this region, of which seven are lysine (K), two are arginine (R), and one is histidine (H), and all these amino acids are located on the surface of the protein (Fig. 4D).

### Removing endogenous heparin reduces the adherence of the key binding region to host cells

Heparinases I and III can respectively degrade sodium heparin or heparin into unsaturated uronic acids. To further test the interaction of the amino acid region 396–458 of the protein encoded by the *Mbov_0510* gene with cell surface heparin, the MDBK cell was used as a model, and the endogenous cell surface heparin was removed using heparinases I and III. Treatment with either heparinase I or III alone had no significant effect on the adhesion of P0510_K_ to MDBK cells (data not shown). However, when both heparinases were co-incubated with MDBK cells, the binding of P0510_K_ to MDBK cells was significantly reduced (Fig. 4E). This result further confirms the role of the amino acid region 396–458 of the Mbov*_0510*-encoded protein in heparin interaction and suggests that this region may have the ability to interact with different types of heparins on host cells.

### *Mbov_0510*-encoded protein conservative analysis

**Fig 5:**
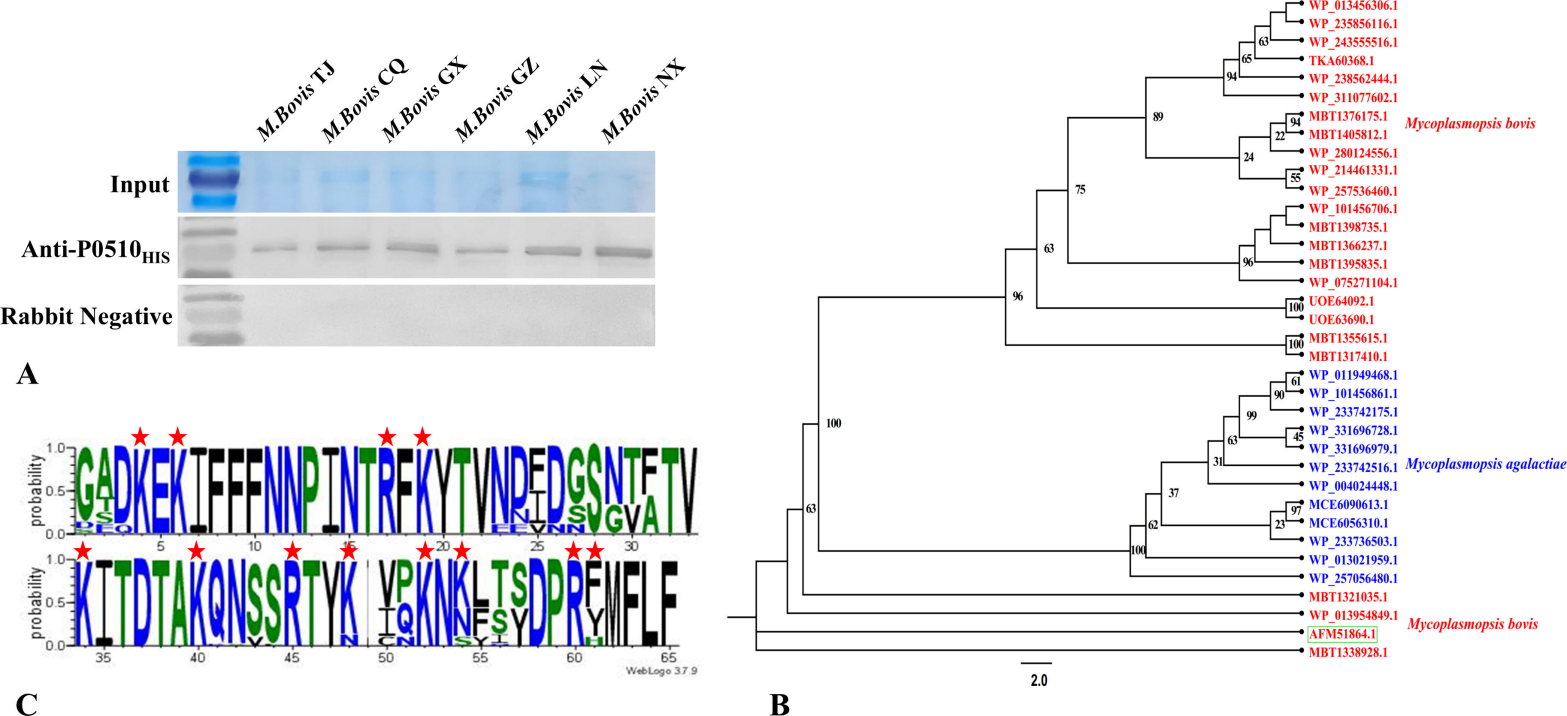
(**A**) The conservation of the protein encoded by *Mbov_0510* was detected using rabbit anti-P0510_HIS_ antibody in *M. bovis* isolates from six different provinces in China. Western blot results showed that the isolates from all six provinces expressed the protein encoded by the *Mbov_0510* gene. (**B**) Phylogenetic tree analysis was performed on homologous proteins of the *Mbov_0510*-encoded protein matched in the NCBI database. The results showed that these proteins were divided into three clusters. In addition to the two clusters of *M. bovis*, *Mycoplasma agalactiae* also expresses homologs of the protein encoded by the *Mbov_0510* gene, forming a separate evolutionary branch. The protein located within the green box in the figure is the protein encoded by the *Mbov_0510* gene. (**C**) Amino acid conservation analysis was conducted on the amino acid region 396-458 of the *Mbov_0510*-encoded protein. The amino acids beneath the red stars are positively charged amino acids, which are highly conserved.

The expression of the *Mbov_0510*-encoded protein in six *M. bovis* strains isolated from six provinces in China was analyzed by western blot. Immunoblot results showed that specific bands could be detected in all test strains using rabbit anti-P0510_HIS_ serum, whereas no bands were detected using pre-immune serum (Fig. 5A). To further investigate the conservation of the *Mbov_0510*-encoded protein, a comprehensive BLAST search was performed on the National Center for Biotechnology Information (NCBI) database for the *Mbov_0510*-encoded protein. The search results included 35 sequences with query cover > 50% and percent identity > 70%. After alignment of the *Mbov_0510*-encoded protein with these 35 sequences using MAFFT, phylogenetic tree analysis was performed using IQ-TREE software. In addition to the *M. bovis*-encoded proteins, 12 *Mycoplasma agalactiae* sequences formed a separate cluster in the phylogenetic tree (Fig. 5B). The amino acid region 396–458 of the *Mbov_0510*-encoded protein was further analyzed using the WebLogo online server (WebLogo 3-About [threeplusone.com]). The results showed that these proteins have highly conserved amino acids, especially the positively charged amino acids (Fig. 5C). This finding suggests that these proteins all have the potential to bind heparin.

## DISCUSSION

*M. bovis* infection can cause a variety of clinical signs, such as pneumonia, arthritis, and mastitis (1, 2). The diversity of clinical signs is often related to the invasiveness of the pathogen into different tissue cells. To date, more than 10 *M. bovis* adhesins have been identified, mainly interacting with host cell surface components. Host cell surface components that have been implicated in *M. bovis* adhesion include plasminogen, fibronectin, amyloid precursor-like protein 2, heparin, annexin A2, collagen IV, vitronectin, and laminin (19, 20, 27). In addition to their role in colonization, host cell-adherent proteins are also involved in *M. bovis* invasion of host cells (19, 20). Therefore, proteins located on the surface of the pathogen that can interact directly with host cells play an important role in pathogen infection. In this study, we found that the protein encoded by the *Mbov_0510* gene of the *M. bovis* HB0801 strain can adhere to EBL and MDBK cells, and this adhesion ability is achieved by binding to heparin on the surface of these two cell types.

As demonstrated in previous studies, *M. bovis* has been isolated from small ruminants, while *Mycoplasma agalactiae* has been isolated from cattle (33–36). It is noteworthy that homologs of the protein encoded by the *Mbov_0510* gene have been identified in *Mycoplasma agalactiae*. It has been demonstrated that this protein binds heparin, a universal component of mammalian cell surfaces, suggesting a potential for significant involvement in the cross-species infection capabilities of both *M.* bovis and M*. agalactiae*. The initial step in achieving the desired niches is effective adherence to target cells, which is followed by access to nutrient conditions that allow growth and multiplication (37). Within these multi-factorial processes, proteins on the mycoplasma surface play a pivotal role in the host-pathogen interaction (37). The identification of surface-localized adhesion-related proteins in *M. bovis* could highlight potential targets for the development of pharmaceuticals, vaccines, and/or immunodiagnostic tests for this economically significant ruminant pathogen (13). In light of the extant research on *M. bovis* adhesins, the prospect of containing *M. bovis* infection and transmission by means of blocking a single adhesin appears to be exceedingly challenging. However, research into novel vaccines, such as peptide-based vaccines, utilizing multiple adhesins, particularly those of significant functional importance, holds considerable potential for advancing the prevention and control of *M. bovis*.

Heparin is involved in the adhesion of several pathogens, such as *Mycoplasma hyopneumoniae*, *Candida albicans*, and SARS-CoV-2 (29–31). Adhesins using heparin as a ligand have also been identified in *M. bovis* (13, 17). Given the distribution characteristics of heparin in host tissues and cells, heparin-interacting adhesins may be closely related to the multi-tissue invasiveness and diverse clinical manifestations of *M. bovis*. In this study, we confirmed the presence of the protein encoded by the *Mbov_*0510 gene on the surface of *M. bovis*. We have also shown that this protein can adhere to host cells. In addition, both naturally infected *M. bovis* serum and serum specific for the protein encoded by the *Mbov_0510* gene significantly blocked the binding of the *Mbov_0510* protein to heparin. However, it is unfortunate that when EBL or MDBK cells were used as models for *M. bovis* infection blocking experiments, no significant blocking effect was observed with antibodies against the *Mbov_0510* protein (data not shown). We speculate that this phenomenon may occur because *M. bovis* possesses multiple adhesins, and the colonization of host cells by *M. bovis* is the result of the synergistic action of these adhesins. Therefore, blocking a single adhesin may have a limited effect on *M. bovis* colonization. Additionally, the antibodies we generated against the *Mbov_0510* protein only partially blocked the binding of the protein to heparin, which may further weaken the role of these antibodies in blocking *M. bovis* adhesion to host cells. Another possibility is that we did not choose an appropriate cell model. The key adhesins of *M. bovis* may differ between different cell models. Studies have shown that when monoclonal antibodies against the VSP protein block the adhesion of *M. bovis*, specific antibodies can block the adhesion of *M. bovis* to EBL cells but do not affect its adhesion to BBE cells (23).

Basic amino acids, such as lysine, arginine, and rarely histidine, are critical for interaction with anionic sulfate or carboxylate groups in heparin through electrostatic and hydrogen bonding. These amino acids can form specific heparin binding motifs such as “XBBXBX,” “XBBBXXBX,” or “XBBBXXBBBXXBBX,” where B is a basic, and X is a hydropathic (neutral and hydrophobic) amino acid residue (38–40). In the heparin-binding critical region that we identified, no classical heparin-binding motifs were detected. However, a large number of basic (positively charged) amino acids were found in this region. Tertiary structure prediction revealed that these positively charged amino acids are all located on the surface of protein. We speculate that the conformational structure composed of multiple positively charged amino acids is involved in heparin binding. Multiple sequence alignment analysis revealed that the key region for heparin binding in the *Mbov_0510*-encoded protein is highly conserved among homologous sequences in the NCBI database, especially the positively charged amino acids, which show high conservation in the matched sequences. This suggests that these proteins may all have the ability to bind heparin. It is important to highlight that, in the phylogenetic tree analysis, both *M. bovis* and *Mycoplasma agalactiae* possess this protein. This suggests that the protein is early evolved.

In the present study, we have identified a heparin-binding protein of *M. bovis* using several approaches, but there are still some limitations. Although we have confirmed that the protein encoded by *Mbov_0510* can adhere to host cells and further demonstrated its binding to heparin on the cell surface, a more direct method to prove the role of this protein in infection would be gene knockout. Unfortunately, because we identified the candidate protein by extracting *M. bovis* membrane proteins and analyzing their interactions with host cells using mass spectrometry, we do not have a corresponding gene knockout strain. Currently, there is no genomic manipulation platform available for *M. bovis* to perform targeted gene knockout, making it impossible to obtain a *Mbov_0510* gene knockout mutant. This leads to an incomplete characterization of the function of the protein. Based on the results of this study, we cannot definitively classify this protein as an *M. bovis* adhesin, but it is reasonable to designate it as a candidate *M.* bovis adhesin.

## MATERIALS AND METHODS

### Bacteria, cells, and culture conditions

The M*. bovis* strains of TJ, CQ, GX, GZ, LN, and NX utilized in this study were maintained in our laboratory. These bacteria were isolated from different regions of China, including Tianjin, Chongqing, Guangxi, Guizhou, Liaoning, and Ningxia. These mycoplasmas were cultivated to the mid-logarithmic growth phase in *M. bovis* broth (PPLO broth medium containing 20% horse serum, 10% yeast extract, and 200 IU/mL penicillin) (1). *Escherichia coli* strains DH5α and BL21(DE3) were cultivated in Luria-Bertani (LB) broth at 37°C (or 16°C) with shaking at 200 rpm/min. LB agar was supplemented with kanamycin (50 µg/mL) or ampicillin (100 µg/mL). The EBL and MDBK cells were cultured in Dulbecco’s modified Eagle medium (DMEM, Gibco) supplemented with 10% inactivated fetal bovine serum (OriCell), 100 U/mL penicillin sodium, and 100 µg/mL streptomycin at 37°C in 5% CO_2_.

### Plasmid construction and protein expression

Genomic DNA from *M. bovis* TJ strain was extracted using a genomic DNA extraction kit (BioFLUX). The MBOV_0510-CF/CR primers, which contain homologous arms for the pET-28a(+) vector, were designed based on *Mbov_0510* gene sequence from *M. bovis* HB0801 strain (GenBank: CP002058.1). The coding sequence of the *Mbov_*0510 lacking a signal peptide was amplified using the primers in Table 1. The target gene was then cloned into the pET-28a(+) vector using the ClonExpress Ultra One Step Cloning Kit (Vazyme). The tryptophan codon “TGA” in *M. bovis*, which functions as a stop codon in *E. coli*, was mutated to “TGG” using the Mut Express II Fast Mutagenesis Kit (Vazyme) and the primers of MBOV_0510-1-F/R, MBOV_0510-2-F/R, MBOV_0510-3-F/R, and MBOV_0510-4&5 F/R (Table 1). The expressed protein was designated P0510_HIS_. The HIS tag of P0510_HIS_ was removed by introducing the LEVLFQGP sequence using primers HVR-3C-F/R, which was then cleaved by HRV-3C protease, yielding protein P0510. To identify the key heparin-binding region, the coding sequence of *Mbov_0510* was serially truncated until the target region was obtained. The region was fused to HIS or GST tags, yielding P0510_K_ and P0510_K_GST_, respectively. The key binding region knockout mutation was obtained by overlap extension PCR, and the recombinant protein was named ΔP0510.

**TABLE 1:** Primers for *MBOV_0510* gene cloning, “TGA” codon mutation, and tag removal

Primer name	Primer sequence (5′−3′)
MBOV_0510-CF	cagcaaatgggtcgcggatccATGGTAAGTGGCAAGGAAAATTTAA
MBOV_0510-CR	acggagctcgaattcggatccTTATCCTTTAAGTCCTAGTAATATTAATATTATTCC
MBOV_0510-1-F	GCAATAGCTGgAGTGATTACATTGTTAAAAAGATTAAGCC
MBOV_0510-1-R	ATCACTcCAGCTATTGCTATTTTGCGGATG
MBOV_0510-2-F	CGATCTATGgAACAGCATTATTATTAAGTATGCTAATAAAATT
MBOV_0510-2-R	TGCTGTTcCATAGATCGGTAAACTTTTTATCAGTAATT
MBOV_0510-3-F	GCCATTTTGgAGCTCATTAGTTAATGCATTAACTGTAGTT
MBOV_0510-3-R	ATGAGCTcCAAAATGGCTGGTTATTTAATTCTG
MBOV_0510-4&5F	gGCAAAAAGTTTTCAATATAGCAAATGgTTTGATGAATATACAGCTAATGTTGCTG
MBOV_0510-4&5R	TATTGAAAACTTTTTGCcCAGTTATTAGCTGATGCTCTAAGTTTTAA
HVR-3C-F	cagcaaatgggtcgcggatccCTGGAAGTTCTGTTCCAGGGGCCCATGGTAAGTGGCAAGGAAAATTTAA
HVR-3C-R	tgcggccgcaagcttgtcgacGGGCCCCTGGAACAGAACTTCCAGTTATCCTTTAAGTCCATAATAAGTTTTCTTT

The recombinant plasmids were transformed into *E. coli* BL21(DE3) cells for protein expression. The transformants were cultivated until the logarithmic phase was attained, then induced with 0.5 mM IPTG at 16°C for 24 h. Recombinant proteins with HIS or GST tags were then purified using Ni-NTA agarose (GE) or glutathione resin columns (Thermo Fisher) in accordance with the manufacturer’s instructions. The protein concentrations were measured using a BCA protein concentration assay kit.

### Preparation and identification of polyclonal antibodies

The purified P0510_HIS_ protein was emulsified with an equal volume of Freund’s complete adjuvant (Sigma) for the initial immunization of Japanese white rabbits by subcutaneous injection at multiple sites at a dose of 200 µg per rabbit. Subsequent booster immunizations were administered at 2-week intervals using the P0510_HIS_ protein mixed with Freund’s incomplete adjuvant (Sigma) at the same dose. Blood samples were collected from the ear veins of the rabbits at 14-day intervals in order to determine the serum antibody titer. This process was continued until the desired level was reached. Once the specified criteria for antibody titer were met, a large volume of blood was collected for serum preparation and stored at −20°C. The antibody titer was determined by ELISA. The specificity of the sera was confirmed by western blot analysis with P0510, 6-GST tag, and an unrelated protein (GenBank: AFM51933.1).

### Whole-cell lysates, membrane, and cytoplasmic protein preparation

*M. bovis* TJ strain in the mid-logarithmic phase was harvested by means of centrifugation at 12,000 × *g* for 10 min, after which the bacterial pellets were washed three times with PBS. The extraction of membrane and cytoplasmic proteins was then conducted using the Proteo Extrat Transmembrane Protein Extraction Kit (Merk) in accordance with the manufacturer’s instructions. The preparation of whole-cell protein from the *M. bovis* was achieved through ultrasonic lysis. The membrane proteins from EBL and MDBK cells were extracted using a membrane protein extraction kit (Solarbio). The concentration of the extracted membrane proteins was determined using a BCA protein concentration assay kit.

### Western blot assay

The western blot was performed with minor modifications to the established protocol (16, 18). ImageJ software (https://imagej.net/ij/) was used for some results analysis.

The antigenicity analysis of *Mbov_0510*-encoded protein was conducted in the following manner: initially, P0520_HIS_ was purified and subjected to SDS-PAGE; thereafter, the protein was transferred to NC film and blocked with 5% gelatin in PBS for 2 h; subsequently, the protein was incubated with 100-fold diluted bovine *M. bovis* sera for 2 h and with HRP-conjugated rabbit anti-bovine IgG secondary antibody (1:8,000) for 1 h; color was developed using a DAB substrate kit.

To analyze the specificity of rabbit anti-P0510_HIS_ serum, the protein P0510, 6-GST tag, and an unrelated protein were run on SDS-PAGE, transferred to an NC film, blocked with 5% gelatin, and incubated with 10,000-fold diluted rabbit anti-P0510_HIS_ serum, followed by 10,000-fold diluted HRP-conjugated goat anti-rabbit antibody. Proteins were visualized with DAB substrate kit.

The distribution and conservation of the protein were analyzed as follows: 2 μg of total-cell, membrane, and cytoplasmic proteins were separated by 12% SDS-PAGE, transferred to an NC film, blocked with 5% gelatin in PBS for 2 h, and incubated with rabbit anti-P0510_HIS_ serum and pre-immune serum (1,000-fold diluted) at room temperature for 2 h, and then with HRP-conjugated goat anti-rabbit IgG antibody (1:5,000) at room temperature for 1 h. Color was developed with a DAB substrate kit.

For heparin binding examination, P0510_HIS_ or P0510 proteins were subjected to 12% SDS-PAGE, transferred to an NC film, blocked as previously described, and incubated with 50 µg/mL heparin-biotin sodium salt, and then with goat anti-biotin peroxidase antibody (2,000-fold diluted). Color was developed with DAB substrate kit. In the serum-blocking heparin-binding assay, the NC membrane was first incubated with *M. bovis*-positive or -negative serum from bovine or rabbit before adding heparin-biotin sodium salt.

### ELISA analysis

The microtiterr plates were coated with 5 µg/mL of purified P0510_HIS_ protein overnight at 4°C for 12 h, then blocked with 5% gelatin in PBS at 37°C for 2 h. Serial twofold dilutions (2^0^ × 5,000 to 2^6^ × 5,000) of rabbit anti-P0510_HIS_ serum and pre-immune serum (100 µL per well) were added and incubated at 37°C for 1 h. Subsequently, 100 µL of 50,0000-fold diluted HRP-conjugated goat anti-rabbit IgG was added and incubated at 37°C for 1 h. Following the addition of TMB and subsequent incubating in the dark for 10 min, the reaction was terminated with 2 M H_₂_SO_₄_. The absorbances were measured at 450 nm to evaluate the antibody titer.

To verify the P0510_HIS_ adhesion to cells, 10 µg/mL cell membrane component was coated onto ELISA plates and blocked as previously described. Different concentrations of purified P0510_HIS_ (25 to 0 µg/mL) were added and incubated at 37°C for 1 h. This was followed by the addition of 100 µL of 2,500-fold diluted rabbit anti-P0510_HIS_ serum and then 100 µL of 12,500-fold diluted peroxidase-conjugated goat anti-rabbit IgG. Color development with TMB and reaction termination with 2 M H₂SO₄ were performed, and OD_450nm_ was measured.

For the analysis of the heparin-binding ability of the *Mbov_0510* gene-encoded protein, 96-well plates were coated with 5 µg/mL purified P0510 and blocked with 5% gelatin. Ten-fold serial dilutions of heparin-biotin sodium salt (10² to 10⁻⁴ µg/mL) were added and incubated at 37°C for 1 h. This was followed by the addition of 5,000-fold diluted goat anti-biotin peroxidase antibody. After adding 50 µL TMB substrate and incubating in the dark at 37°C for 10 min, the reaction was terminated with 2 M H_₂_SO_₄_. The OD_450nm_ absorbance was then measured.

### Dot blot analysis

The proteins were fixed onto the NC membrane without denaturation, and the NC film was sealed with 5% gelatin at room temperature for 2 h. The film was then incubated with biotinylated heparin (50 µg/mL) at room temperature for 2 h. The peroxidase-conjugate goat anti-biotin antibodies (1:2,000) were employed for the detection of the binding heparin at room temperature for 1 h. Finally, the color was developed with a DAB substrate kit. In order to confirm the successful printing of the protein onto the NC membrane, an input control with Ponceau S staining was established.

### Indirect immunofluorescence assay

To investigate the surface distribution of the *Mbov_0510*-encoded protein on *M. bovis*, IFA was performed as previously described (16). Fresh *M. bovis* TJ strain pellets were washed and incubated with 500-fold diluted rabbit anti-P0510_HIS_ immune serum or pre-immune serum at 37°C for 1 h. After washing and centrifugation, the pellets were resuspended in 100-fold diluted fluorescein-conjugated goat anti-rabbit IgG (H + L) antibody (Zhongshan Golden Bridge) and incubated at 37°C for 1 h. Fluorescence was observed under an inverted fluorescence microscope (Zeiss Axio Observer 5).

To determine the directed adhesion of the *MBOV_0510*-encoded protein to host cells, EBL/MDBK cells were cultured for 24 h, washed, and fixed with 4% paraformaldehyde. The cells were blocked with 1% BSA in PBS at 37°C for 2 h, washed, and incubated with 100 µg/mL P0510 protein or PBS (control) at 37°C for 1 h. Rabbit anti-P0510_HIS_ serum (1000-fold diluted) was added, followed by 100-fold diluted fluorescein-conjugated goat anti-rabbit IgG (H + L) (ZSGB-BIO) in the dark at 37°C for 1 h. Cell nuclei were stained with DAPI, and fluorescence was observed under a laser confocal microscope (LSM880-ZEISS).

To analyze the effect of heparinase digestion on the adhesion of *MBOV_0510*-encoded protein, MDBK cells were cultured, washed, and fixed. Heparinases I and III were utilized to digest the cells at 30°C for 24 h. After digestion, the cells were blocked with 1% BSA as previously described, washed, and incubated with 100 µg/mL of the P0510_K_ protein. The cells were incubated with rabbit anti-P0510_HIS_ serum (1,000-fold diluted) at 37°C for 1 h, followed by an incubation with fluorescein-conjugated goat anti-rabbit IgG (H + L) (ZSGB-BIO) (100-fold diluted) in the dark at 37°C for 1 h. The fluorescence was observed under an inverted fluorescence microscope.

### Tertiary structure prediction and conservation analysis

The SWISS-MODEL online server (https://swissmodel.expasy.org) was employed for tertiary structural prediction of the *Mbov_0510*-encoded protein.

The conservation of the protein encoded by *Mbov_0510* was analyzed using the NCBI database. First, the BLAST module was employed to search for homologous sequences of the *Mbov_0510*-encoded protein in the non-redundant protein sequences (nr) database. Sequences with query cover > 50% and percent identity > 70% were retained for further analysis. The sequences of the *Mbov_0510*-encoded protein and the selected homologous proteins were then aligned using the MAFFT software (version 7) (41). The aligned sequences were then submitted to the Gblocks online server for trimming (42), and a phylogenetic tree was constructed using the IQ-tree ML tool (43, 44). In addition, the conservation of amino acids in the key region of the *Mbov_0510*-encoded protein that binds to heparin was further analyzed using the WebLogo online server (https://weblogo.berkeley.edu/logo.cgi) (45).

## Data Availability

All data supporting the findings of this study are available from the corresponding author on reasonable request.
